# Phenyl *N*-(4-fluorophen­yl)carbamate

**DOI:** 10.1107/S1600536809013312

**Published:** 2009-04-18

**Authors:** Zhao Yang, Zhi-Xiang Wang

**Affiliations:** a Department of Pharmaceutical Engineering, China Pharmaceutical University, Tongjiaxiang No. 24 Nanjing, Nanjing 210009, People’s Republic of China

## Abstract

The asymmetric unit of the title compound, C_13_H_10_FNO_2_, contains two crystallographically independent mol­ecules. The aromatic rings are oriented at dihedral angles of 61.77 (3) and 53.94 (3)° in the two mol­ecules. An N—H⋯O hydrogen bond links the mol­ecules. In the crystal structure, inter­molecular N—H⋯O hydrogen bonds link the mol­ecules into chains. Weak C—H⋯π inter­actions are also present.

## Related literature

For a related structure, see: Hynes *et al.* (2008 ▶). For bond-length data, see: Allen *et al.* (1987 ▶).
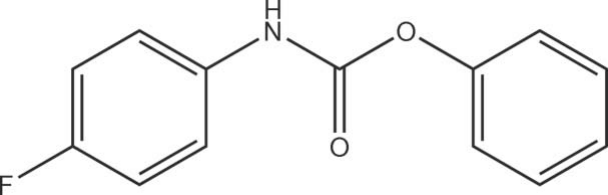


## Experimental

### 

#### Crystal data

C_13_H_10_FNO_2_
*M* *_r_* = 231.22
Triclinic, 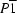

*a* = 5.8860 (12) Å
*b* = 7.8540 (16) Å
*c* = 24.761 (5) Å
α = 96.62 (3)°
β = 92.82 (3)°
γ = 91.19 (3)°
*V* = 1135.3 (4) Å^3^
*Z* = 4
Mo *K*α radiation
μ = 0.10 mm^−1^
*T* = 294 K
0.10 × 0.10 × 0.08 mm

#### Data collection

Enraf–Nonius CAD-4 diffractometer
Absorption correction: ψ scan (North *et al.*, 1968 ▶) *T*_min_ = 0.990, *T*_max_ = 0.992
4548 measured reflections
4110 independent reflections
1662 reflections with *I* > 2σ(*I*)
*R*_int_ = 0.060
3 standard reflections frequency: 120 min intensity decay: 1%

#### Refinement

*R*[*F* ^2^ > 2σ(*F* ^2^)] = 0.073
*wR*(*F* ^2^) = 0.157
*S* = 1.00
4110 reflections
307 parameters
H-atom parameters constrained
Δρ_max_ = 0.13 e Å^−3^
Δρ_min_ = −0.13 e Å^−3^

### 

Data collection: *CAD-4 Software* (Enraf–Nonius, 1989 ▶); cell refinement: *CAD-4 Software*; data reduction: *XCAD4* (Harms & Wocadlo, 1995 ▶); program(s) used to solve structure: *SHELXS97* (Sheldrick, 2008 ▶); program(s) used to refine structure: *SHELXL97* (Sheldrick, 2008 ▶); molecular graphics: *PLATON* (Spek, 2009 ▶); software used to prepare material for publication: *SHELXTL* (Sheldrick, 2008 ▶).

## Supplementary Material

Crystal structure: contains datablocks global, I. DOI: 10.1107/S1600536809013312/hk2663sup1.cif

Structure factors: contains datablocks I. DOI: 10.1107/S1600536809013312/hk2663Isup2.hkl

Additional supplementary materials:  crystallographic information; 3D view; checkCIF report

## Figures and Tables

**Table 1:** Hydrogen-bond geometry (Å, °)

*D*—H⋯*A*	*D*—H	H⋯*A*	*D*⋯*A*	*D*—H⋯*A*
N1—H1*A*⋯O4^i^	0.86	2.32	3.044 (4)	142
N2—H2*C*⋯O2	0.86	2.08	2.931 (4)	171
C19—H19*A*⋯*Cg*2^ii^	0.93	2.94	3.644 (4)	134
C23—H23*A*⋯*Cg*1^iii^	0.93	2.97	3.710 (5)	138

Symmetry codes: (i) 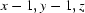; (ii) 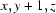; (iii) 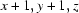. *Cg*1 and *Cg*2 are the centroids of the C1–C6 and C8–C13 rings, respectively.

**Fig. 1.**
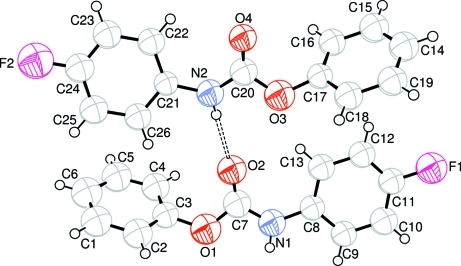
The molecular structure of the title molecule, with the atom-numbering scheme. Hydrogen bond is shown as dashed line.

**Fig. 2.**
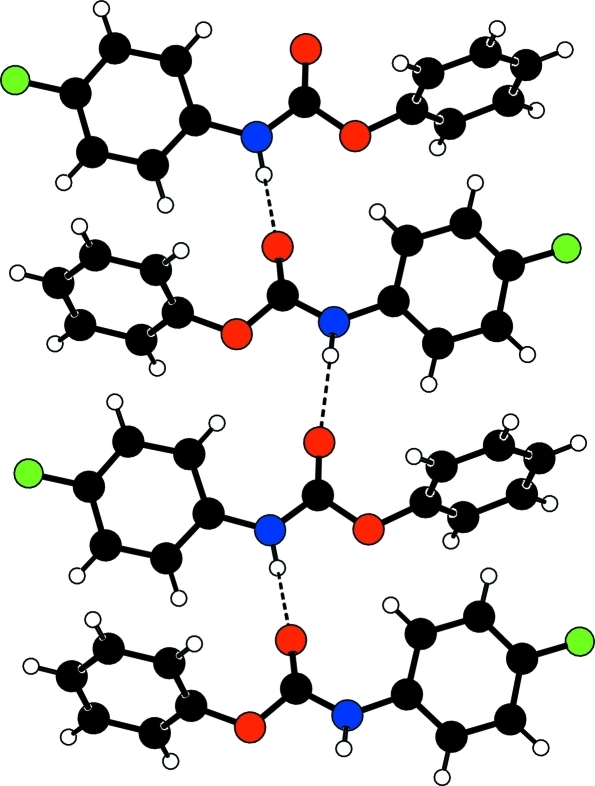
A partial packing diagram of the title compound. Hydrogen bonds are shown as dashed lines.

## supplementary crystallographic information

### Comment

Some derivatives of aniline are important chemical materials. We report herein the crystal structure of the title compound.

The asymmetric unit of the title compound contains two crystallographically independent molecules (Fig. 1), in which the bond lengths (Allen *et al.*, 1987) and angles are within normal ranges. Rings A (C1-C6), B (C8-C13) and A' (C14-C19), B' (C21-C26) are, of course, planar, and they are oriented at dihedral angles of A/B = 61.77 (3) and A'/B' = 53.94 (3) °. Intramolecular N-H···O hydrogen bond (Table 1) links the molecules.

In the crystal structure, intra- and intermolecular N-H···O hydrogen bonds (Table 1) link the molecules into chains (Fig. 2), in which they may be effective in the stabilization of the structure. There also exist weak C—H···π interactions (Table 1).

### Experimental

For the preparation of the title compound, phenyl chloroformate (1.0 ml) was added slowly to a cold solution of 4-fluorbenzenamine (1.0 g) and triethylamine (0.8 ml) in methylene chloride (10 ml) at 273 K. The mixture was then warmed and stirred for 1 h at room temperature. It was washed with water (20 ml), dried and concentrated to give the title compound (yield; 1.3 g) (Hynes *et al.*,  2008). Crystals suitable for X-ray analysis were obtained by slow evaporation of a methanol solution.

### Refinement

H atoms were positioned geometrically, with N-H = 0.86 Å (for NH) and C-H = 0.93 Å for aromatic H and constrained to ride on their parent atoms, with U_iso_(H) = 1.2U_eq_(C,N).

### Crystal data

**Table:**

C_13_H_10_FNO_2_	*Z* = 4
*M**_r_* = 231.22	*F*(000) = 480
Triclinic, *P*1	*D*_x_ = 1.353 Mg m^−^^3^
Hall symbol: -P 1	Mo *K*α radiation, λ = 0.71073 Å
*a* = 5.8860 (12) Å	Cell parameters from 25 reflections
*b* = 7.8540 (16) Å	θ = 8–12°
*c* = 24.761 (5) Å	µ = 0.10 mm^−^^1^
α = 96.62 (3)°	*T* = 294 K
β = 92.82 (3)°	Block, colorless
γ = 91.19 (3)°	0.10 × 0.10 × 0.08 mm
*V* = 1135.3 (4) Å^3^	

### Data collection

**Table:**

Enraf–Nonius CAD-4 diffractometer	1662 reflections with *I* > 2σ(*I*)
Radiation source: fine-focus sealed tube	*R*_int_ = 0.060
graphite	θ_max_ = 25.3°, θ_min_ = 1.7°
ω/2θ scans	*h* = 0→7
Absorption correction: ψ scan (North *et al.*, 1968)	*k* = −9→9
*T*_min_ = 0.990, *T*_max_ = 0.992	*l* = −29→29
4548 measured reflections	3 standard reflections every 120 min
4110 independent reflections	intensity decay: 1%

### Refinement

**Table:**

Refinement on *F*^2^	Primary atom site location: structure-invariant direct methods
Least-squares matrix: full	Secondary atom site location: difference Fourier map
*R*[*F*^2^ > 2σ(*F*^2^)] = 0.073	Hydrogen site location: inferred from neighbouring sites
*wR*(*F*^2^) = 0.157	H-atom parameters constrained
*S* = 1.00	*w* = 1/[σ^2^(*F*_o_^2^) + (0.052*P*)^2^] where *P* = (*F*_o_^2^ + 2*F*_c_^2^)/3
4110 reflections	(Δ/σ)_max_ < 0.001
307 parameters	Δρ_max_ = 0.13 e Å^−^^3^
0 restraints	Δρ_min_ = −0.13 e Å^−^^3^

### Special details

**Table:**

Geometry. All e.s.d.'s (except the e.s.d. in the dihedral angle between two l.s. planes) are estimated using the full covariance matrix. The cell e.s.d.'s are taken into account individually in the estimation of e.s.d.'s in distances, angles and torsion angles; correlations between e.s.d.'s in cell parameters are only used when they are defined by crystal symmetry. An approximate (isotropic) treatment of cell e.s.d.'s is used for estimating e.s.d.'s involving l.s. planes.
Refinement. Refinement of *F*^2^ against ALL reflections. The weighted *R*-factor *wR* and goodness of fit *S* are based on *F*^2^, conventional *R*-factors *R* are based on *F*, with *F* set to zero for negative *F*^2^. The threshold expression of *F*^2^ > σ(*F*^2^) is used only for calculating *R*-factors(gt) *etc*. and is not relevant to the choice of reflections for refinement. *R*-factors based on *F*^2^ are statistically about twice as large as those based on *F*, and *R*- factors based on ALL data will be even larger.

### Fractional atomic coordinates and isotropic or equivalent isotropic displacement parameters (Å^2^)

**Table:**

	*x*	*y*	*z*	*U*_iso_*/*U*_eq_	
N1	0.0390 (6)	−0.6473 (4)	0.26993 (14)	0.0831 (11)	
H1A	−0.0691	−0.7213	0.2599	0.100*	
N2	0.6939 (5)	−0.2644 (4)	0.22504 (13)	0.0755 (11)	
H2C	0.6083	−0.3507	0.2300	0.091*	
F1	−0.0366 (5)	−0.3165 (3)	0.47653 (11)	0.1065 (9)	
F2	1.1428 (5)	−0.3284 (4)	0.03482 (12)	0.1253 (11)	
O1	0.1510 (5)	−0.7508 (4)	0.19019 (13)	0.1045 (12)	
O2	0.3575 (5)	−0.5355 (4)	0.23766 (11)	0.0916 (10)	
O3	0.5300 (5)	−0.1536 (4)	0.29759 (12)	0.0938 (11)	
O4	0.8164 (4)	−0.0021 (3)	0.26628 (10)	0.0724 (8)	
C1	0.3835 (12)	−0.7830 (7)	0.0572 (2)	0.1033 (18)	
H1B	0.3416	−0.7637	0.0218	0.124*	
C2	0.2332 (9)	−0.7392 (6)	0.0984 (3)	0.0934 (16)	
H2A	0.0941	−0.6896	0.0917	0.112*	
C3	0.3045 (9)	−0.7740 (6)	0.1487 (2)	0.0763 (14)	
C4	0.5077 (11)	−0.8420 (6)	0.1599 (2)	0.0894 (16)	
H4A	0.5516	−0.8605	0.1953	0.107*	
C5	0.6465 (9)	−0.8828 (6)	0.1186 (3)	0.1024 (18)	
H5A	0.7849	−0.9327	0.1259	0.123*	
C6	0.5889 (11)	−0.8528 (7)	0.0673 (3)	0.108 (2)	
H6A	0.6871	−0.8792	0.0394	0.130*	
C7	0.1980 (8)	−0.6340 (6)	0.23447 (19)	0.0860 (15)	
C8	0.0241 (7)	−0.5556 (5)	0.32213 (17)	0.0698 (12)	
C9	−0.1636 (7)	−0.5849 (5)	0.35086 (18)	0.0740 (12)	
H9A	−0.2782	−0.6603	0.3349	0.089*	
C10	−0.1859 (8)	−0.5051 (6)	0.4028 (2)	0.0861 (14)	
H10A	−0.3134	−0.5253	0.4221	0.103*	
C11	−0.0157 (8)	−0.3963 (5)	0.42483 (19)	0.0776 (13)	
C12	0.1691 (8)	−0.3626 (5)	0.39833 (18)	0.0772 (13)	
H12A	0.2818	−0.2868	0.4150	0.093*	
C13	0.1923 (7)	−0.4407 (5)	0.34595 (18)	0.0784 (13)	
H13A	0.3192	−0.4165	0.3269	0.094*	
C14	0.4753 (10)	0.1478 (6)	0.4438 (2)	0.0860 (15)	
H14A	0.4594	0.2114	0.4774	0.103*	
C15	0.6668 (9)	0.0580 (6)	0.4346 (2)	0.0882 (15)	
H15A	0.7815	0.0618	0.4620	0.106*	
C16	0.6942 (7)	−0.0391 (5)	0.3851 (2)	0.0744 (13)	
H16A	0.8265	−0.0994	0.3790	0.089*	
C17	0.5254 (8)	−0.0445 (5)	0.34616 (18)	0.0647 (11)	
C18	0.3329 (8)	0.0490 (6)	0.35436 (18)	0.0775 (13)	
H18A	0.2203	0.0478	0.3266	0.093*	
C19	0.3070 (9)	0.1455 (6)	0.4044 (2)	0.0829 (14)	
H19A	0.1761	0.2075	0.4107	0.099*	
C20	0.6969 (7)	−0.1294 (6)	0.26345 (17)	0.0763 (13)	
C21	0.8171 (7)	−0.2801 (5)	0.17706 (16)	0.0588 (10)	
C22	1.0269 (7)	−0.2011 (5)	0.17460 (18)	0.0738 (12)	
H22A	1.0954	−0.1378	0.2054	0.089*	
C23	1.1343 (7)	−0.2167 (5)	0.12616 (19)	0.0798 (13)	
H23A	1.2738	−0.1612	0.1237	0.096*	
C24	1.0365 (9)	−0.3124 (6)	0.08250 (19)	0.0840 (14)	
C25	0.8320 (8)	−0.3995 (5)	0.08437 (18)	0.0813 (13)	
H25A	0.7693	−0.4681	0.0540	0.098*	
C26	0.7227 (7)	−0.3818 (5)	0.13273 (17)	0.0717 (12)	
H26A	0.5845	−0.4393	0.1352	0.086*	

### Atomic displacement parameters (Å^2^)

**Table:**

	*U*^11^	*U*^22^	*U*^33^	*U*^12^	*U*^13^	*U*^23^
N1	0.078 (3)	0.080 (3)	0.086 (3)	−0.031 (2)	0.023 (2)	−0.013 (2)
N2	0.082 (3)	0.062 (2)	0.080 (3)	−0.038 (2)	0.023 (2)	−0.0024 (19)
F1	0.120 (2)	0.102 (2)	0.094 (2)	−0.0065 (17)	0.0279 (17)	−0.0139 (16)
F2	0.140 (3)	0.121 (2)	0.112 (2)	−0.0283 (19)	0.061 (2)	−0.0174 (18)
O1	0.105 (3)	0.109 (3)	0.091 (2)	−0.056 (2)	0.030 (2)	−0.027 (2)
O2	0.094 (2)	0.085 (2)	0.091 (2)	−0.0490 (18)	0.0269 (18)	−0.0119 (17)
O3	0.099 (2)	0.086 (2)	0.089 (2)	−0.0449 (18)	0.0397 (19)	−0.0265 (18)
O4	0.0745 (19)	0.0645 (18)	0.0752 (19)	−0.0264 (15)	0.0099 (15)	−0.0018 (14)
C1	0.112 (5)	0.125 (5)	0.071 (4)	−0.036 (4)	−0.012 (4)	0.014 (3)
C2	0.081 (4)	0.089 (4)	0.112 (5)	−0.014 (3)	−0.010 (4)	0.030 (3)
C3	0.065 (3)	0.070 (3)	0.090 (4)	−0.027 (3)	0.014 (3)	−0.005 (3)
C4	0.099 (4)	0.083 (4)	0.083 (4)	−0.035 (3)	−0.008 (3)	0.008 (3)
C5	0.080 (4)	0.063 (3)	0.163 (6)	−0.007 (3)	−0.009 (5)	0.013 (4)
C6	0.081 (4)	0.102 (5)	0.134 (6)	−0.029 (4)	0.032 (4)	−0.025 (4)
C7	0.081 (4)	0.093 (4)	0.077 (3)	−0.033 (3)	0.015 (3)	−0.015 (3)
C8	0.069 (3)	0.062 (3)	0.079 (3)	−0.017 (2)	0.015 (2)	0.008 (2)
C9	0.066 (3)	0.067 (3)	0.087 (3)	−0.016 (2)	0.010 (2)	0.002 (2)
C10	0.083 (3)	0.078 (3)	0.097 (4)	−0.013 (3)	0.025 (3)	0.006 (3)
C11	0.081 (4)	0.061 (3)	0.089 (4)	0.005 (3)	0.024 (3)	−0.007 (3)
C12	0.081 (3)	0.074 (3)	0.076 (3)	−0.015 (3)	0.000 (3)	0.009 (3)
C13	0.075 (3)	0.077 (3)	0.082 (3)	−0.030 (2)	0.009 (2)	0.006 (3)
C14	0.093 (4)	0.086 (4)	0.078 (4)	−0.010 (3)	0.020 (3)	0.002 (3)
C15	0.090 (4)	0.098 (4)	0.077 (4)	−0.012 (3)	0.002 (3)	0.018 (3)
C16	0.055 (3)	0.064 (3)	0.108 (4)	−0.001 (2)	0.015 (3)	0.020 (3)
C17	0.065 (3)	0.057 (3)	0.072 (3)	−0.014 (2)	0.012 (3)	0.005 (2)
C18	0.077 (4)	0.078 (3)	0.079 (3)	−0.020 (3)	−0.004 (3)	0.021 (3)
C19	0.080 (4)	0.069 (3)	0.102 (4)	0.013 (3)	0.022 (3)	0.008 (3)
C20	0.081 (3)	0.070 (3)	0.076 (3)	−0.028 (3)	0.017 (3)	−0.003 (3)
C21	0.059 (3)	0.048 (2)	0.068 (3)	−0.007 (2)	0.003 (2)	0.002 (2)
C22	0.054 (3)	0.073 (3)	0.090 (3)	−0.018 (2)	0.002 (2)	−0.005 (2)
C23	0.068 (3)	0.070 (3)	0.099 (4)	−0.010 (2)	0.029 (3)	−0.007 (3)
C24	0.099 (4)	0.070 (3)	0.082 (4)	−0.006 (3)	0.032 (3)	−0.006 (3)
C25	0.093 (4)	0.073 (3)	0.074 (3)	−0.006 (3)	0.009 (3)	−0.008 (2)
C26	0.072 (3)	0.065 (3)	0.074 (3)	−0.018 (2)	0.007 (2)	−0.007 (2)

### Geometric parameters (Å, °)

**Table:**

N1—C7	1.325 (5)	C9—H9A	0.9300
N1—C8	1.413 (4)	C10—C11	1.354 (5)
N1—H1A	0.8600	C10—H10A	0.9300
N2—C20	1.340 (4)	C11—C12	1.335 (5)
N2—C21	1.417 (4)	C12—C13	1.384 (5)
N2—H2C	0.8600	C12—H12A	0.9300
F1—C11	1.371 (4)	C13—H13A	0.9300
F2—C24	1.358 (4)	C14—C19	1.354 (6)
O1—C3	1.399 (5)	C14—C15	1.357 (6)
O1—C7	1.359 (4)	C14—H14A	0.9300
O2—C7	1.198 (4)	C15—C16	1.385 (6)
O3—C17	1.396 (5)	C15—H15A	0.9300
O3—C20	1.351 (4)	C16—C17	1.347 (5)
O4—C20	1.204 (4)	C16—H16A	0.9300
C1—C6	1.361 (7)	C17—C18	1.374 (5)
C1—C2	1.400 (6)	C18—C19	1.393 (6)
C1—H1B	0.9300	C18—H18A	0.9300
C2—C3	1.355 (6)	C19—H19A	0.9300
C2—H2A	0.9300	C21—C26	1.368 (5)
C3—C4	1.349 (6)	C21—C22	1.377 (5)
C4—C5	1.353 (6)	C22—C23	1.377 (5)
C4—H4A	0.9300	C22—H22A	0.9300
C5—C6	1.348 (7)	C23—C24	1.341 (5)
C5—H5A	0.9300	C23—H23A	0.9300
C6—H6A	0.9300	C24—C25	1.377 (6)
C8—C9	1.373 (5)	C25—C26	1.381 (5)
C8—C13	1.388 (5)	C25—H25A	0.9300
C9—C10	1.379 (5)	C26—H26A	0.9300
			
C7—N1—C8	128.3 (4)	C13—C12—H12A	120.1
C7—N1—H1A	115.8	C12—C13—C8	119.2 (4)
C8—N1—H1A	115.8	C12—C13—H13A	120.4
C20—N2—C21	126.8 (3)	C8—C13—H13A	120.4
C20—N2—H2C	116.6	C19—C14—C15	120.5 (5)
C21—N2—H2C	116.6	C19—C14—H14A	119.7
C7—O1—C3	120.4 (3)	C15—C14—H14A	119.7
C20—O3—C17	118.5 (3)	C14—C15—C16	121.0 (5)
C6—C1—C2	122.3 (5)	C14—C15—H15A	119.5
C6—C1—H1B	118.9	C16—C15—H15A	119.5
C2—C1—H1B	118.9	C17—C16—C15	118.7 (4)
C3—C2—C1	115.4 (5)	C17—C16—H16A	120.6
C3—C2—H2A	122.3	C15—C16—H16A	120.6
C1—C2—H2A	122.3	C16—C17—C18	120.9 (4)
C4—C3—C2	123.5 (5)	C16—C17—O3	121.9 (5)
C4—C3—O1	118.5 (6)	C18—C17—O3	117.0 (4)
C2—C3—O1	117.8 (6)	C17—C18—C19	119.7 (4)
C3—C4—C5	118.9 (5)	C17—C18—H18A	120.1
C3—C4—H4A	120.6	C19—C18—H18A	120.1
C5—C4—H4A	120.6	C14—C19—C18	119.0 (5)
C6—C5—C4	121.5 (6)	C14—C19—H19A	120.5
C6—C5—H5A	119.2	C18—C19—H19A	120.5
C4—C5—H5A	119.2	O4—C20—N2	127.1 (4)
C5—C6—C1	118.4 (6)	O4—C20—O3	124.4 (4)
C5—C6—H6A	120.8	N2—C20—O3	108.3 (3)
C1—C6—H6A	120.8	C26—C21—C22	120.3 (4)
O2—C7—N1	128.5 (4)	C26—C21—N2	117.4 (3)
O2—C7—O1	122.5 (4)	C22—C21—N2	122.3 (4)
N1—C7—O1	109.0 (4)	C21—C22—C23	119.4 (4)
C9—C8—C13	118.8 (4)	C21—C22—H22A	120.3
C9—C8—N1	118.1 (4)	C23—C22—H22A	120.3
C13—C8—N1	123.0 (4)	C24—C23—C22	119.6 (4)
C8—C9—C10	121.4 (4)	C24—C23—H23A	120.2
C8—C9—H9A	119.3	C22—C23—H23A	120.2
C10—C9—H9A	119.3	C23—C24—F2	119.6 (4)
C11—C10—C9	117.7 (4)	C23—C24—C25	122.3 (4)
C11—C10—H10A	121.2	F2—C24—C25	118.1 (4)
C9—C10—H10A	121.2	C24—C25—C26	118.1 (4)
C12—C11—C10	123.0 (4)	C24—C25—H25A	121.0
C12—C11—F1	118.9 (4)	C26—C25—H25A	121.0
C10—C11—F1	118.1 (4)	C21—C26—C25	120.2 (4)
C11—C12—C13	119.8 (4)	C21—C26—H26A	119.9
C11—C12—H12A	120.1	C25—C26—H26A	119.9
			
C6—C1—C2—C3	−1.2 (7)	C19—C14—C15—C16	−0.6 (7)
C1—C2—C3—C4	1.7 (7)	C14—C15—C16—C17	−0.7 (6)
C1—C2—C3—O1	−173.0 (4)	C15—C16—C17—C18	2.3 (6)
C7—O1—C3—C4	67.3 (6)	C15—C16—C17—O3	−173.0 (3)
C7—O1—C3—C2	−117.7 (5)	C20—O3—C17—C16	−63.8 (6)
C2—C3—C4—C5	−2.0 (7)	C20—O3—C17—C18	120.8 (4)
O1—C3—C4—C5	172.7 (4)	C16—C17—C18—C19	−2.6 (6)
C3—C4—C5—C6	1.8 (7)	O3—C17—C18—C19	172.9 (4)
C4—C5—C6—C1	−1.3 (8)	C15—C14—C19—C18	0.3 (7)
C2—C1—C6—C5	1.1 (8)	C17—C18—C19—C14	1.3 (6)
C8—N1—C7—O2	−4.2 (9)	C21—N2—C20—O4	−5.8 (8)
C8—N1—C7—O1	177.7 (4)	C21—N2—C20—O3	170.2 (4)
C3—O1—C7—O2	7.6 (8)	C17—O3—C20—O4	−15.0 (7)
C3—O1—C7—N1	−174.2 (4)	C17—O3—C20—N2	168.9 (4)
C7—N1—C8—C9	176.8 (5)	C20—N2—C21—C26	−150.6 (4)
C7—N1—C8—C13	−4.7 (7)	C20—N2—C21—C22	31.2 (6)
C13—C8—C9—C10	−1.1 (7)	C26—C21—C22—C23	4.0 (6)
N1—C8—C9—C10	177.5 (4)	N2—C21—C22—C23	−177.8 (4)
C8—C9—C10—C11	−0.1 (7)	C21—C22—C23—C24	−1.9 (7)
C9—C10—C11—C12	0.6 (7)	C22—C23—C24—F2	−179.7 (4)
C9—C10—C11—F1	−180.0 (4)	C22—C23—C24—C25	−1.2 (8)
C10—C11—C12—C13	0.0 (8)	C23—C24—C25—C26	2.1 (7)
F1—C11—C12—C13	−179.4 (4)	F2—C24—C25—C26	−179.4 (4)
C11—C12—C13—C8	−1.1 (7)	C22—C21—C26—C25	−3.1 (6)
C9—C8—C13—C12	1.6 (7)	N2—C21—C26—C25	178.6 (4)
N1—C8—C13—C12	−176.9 (4)	C24—C25—C26—C21	0.1 (7)

### Hydrogen-bond geometry (Å, °)

**Table:**

*D*—H···*A*	*D*—H	H···*A*	*D*···*A*	*D*—H···*A*
N1—H1A···O4^i^	0.86	2.32	3.044 (4)	142
N2—H2C···O2	0.86	2.08	2.931 (4)	171
C19—H19A···Cg2^ii^	0.93	2.94	3.644 (4)	134
C23—H23A···Cg1^iii^	0.93	2.97	3.710 (5)	138

Symmetry codes: (i) *x*−1, *y*−1, *z*; (ii) *x*, *y*+1, *z*; (iii) *x*+1, *y*+1, *z*.

## References

[bb1] Allen, F. H., Kennard, O., Watson, D. G., Brammer, L., Orpen, A. G. & Taylor, R. (1987). *J. Chem. Soc. Perkin Trans. 2*, pp. S1–19.

[bb2] Enraf–Nonius (1989). *CAD-4 Software* Enraf–Nonius, Delft. The Netherlands.

[bb3] Harms, K. & Wocadlo, S. (1995). *XCAD4* University of Marburg, Germany.

[bb4] Hynes, J. J., Dyckman, A. J., Lin, S. W. & Stephen, T. (2008). *J. Med. Chem.* **51**, 4–16.

[bb5] North, A. C. T., Phillips, D. C. & Mathews, F. S. (1968). *Acta Cryst.* A**24**, 351–359.

[bb6] Sheldrick, G. M. (2008). *Acta Cryst.* A**64**, 112–122. 10.1107/S0108767307043930 18156677

[bb7] Spek, A. L. (2009). *Acta Cryst.* D**65**, 148–155. 10.1107/S090744490804362X PMC2631630 19171970

